# Impact of green innovation on carbon reduction in China

**DOI:** 10.1038/s41598-024-64554-y

**Published:** 2024-06-18

**Authors:** Haiyan Shan, Shangmiao Shao

**Affiliations:** School of Management Science and Engineering, Nanjing University of Information Science and Technology, Nanjing, 210044 China

**Keywords:** Green innovation, Carbon emission, Big data, Mediating effect, Environmental impact, Sustainability

## Abstract

Green innovation directly encompasses the two major concepts of green and innovation in the new development concepts, which provides a powerful driving force to support Chinese-style modernisation. This paper empirically tests the relationship between green innovation and carbon emission intensity using a double fixed effects model. Based on the panel data of 30 provinces in China, the mediation effect model of “green innovation-big data-carbon emission” is constructed. The result shows that green innovation has a noticeable direct negative effect on urban carbon emission intensity. The conclusions are robust after considering measurement errors and endogenous problems. Furthermore, it is found that big data plays a significant role in strengthening the relationship between green innovation and carbon emission intensity. The findings in this study not only advance the study on green innovation and carbon emissions but also provide a new perspective on the role of big data.

Global warming persists as a pressing challenge for humanity. As countries and economies progress, energy consumption remains pivotal, often fueling a detrimental cycle between development and pollution. Therefore, prioritising the advancement of cutting-edge technologies and enhancing resource efficiency are imperative for fostering sustainability and mitigating environmental challenges. Furthermore, emerging technologies, such as digitalisation, play a crucial role in this endeavour. Also, green development emerges as a prominent trend globally, underscored by its integration into our 13th Five-Year Plan as a guiding principle. In the realm of data compilation methodologies, the scientific and technological revolution has ushered in the era of big data, characterised by its 5V attributes—velocity, volume, value, variety, and veracity—significantly impacting the manufacturing sector, which is the cornerstone of our economy. In the digital economy era marked by exponential technological growth, enterprises gain a competitive advantage through the exploitation of asymmetric information barriers^1^. Hence, the relentless advancement of big data technology mandates enterprises to undergo digital and intelligent transformations to optimise resource allocation and enhance production efficiency, thereby facilitating high-quality and green development^2^.

While the manufacturing industry’s development has bolstered national power, it also brings forth consequential challenges that demand attention. Despite notable achievements, the growth of Chinese industry has led to resource depletion and environmental degradation, with carbon emissions emerging as a significant contributor to industrial pollution, drawing global concern. Since 2020, China has set ambitious targets for carbon peaking and neutrality, making sustainable development a paramount objective to achieve these dual carbon goals. Besides, essential to this endeavour is green technological innovation, which holds the key to fostering green development by optimising resource utilisation and curbing pollutant production. As manufacturing companies increasingly undergo digital and intelligent transformations, there arises a pertinent inquiry into the potential impact of big data technology on green technological innovation and its underlying determinants, a topic of scholarly discussion^3–5^. Yet, scant attention has been paid to exploring whether green innovation has an impact on big data technologies. This paper explores the impact of green innovation influences big data technologies.

This study therefore pioneers the examination of the interaction between green innovation and big data technologies. Through comprehensive research, we uncover that big data technology assumes an active role rather than merely observing the interplay between green innovation and carbon emissions. Taken together, it emerges as a dynamic participant, influenced by green innovation and subsequently driving toward a more positive trajectory. Building on these insights, our investigation delves deeper into the intricate relationship between carbon emissions, green innovation, and big data. Our findings illuminate the significant role of big data in bridging the gap between carbon emissions and green innovation, revealing its mediating effect in curbing carbon intensity through rigorous data analysis and research. Thus, this study not only enhances our comprehension of the nexus that exists between green innovation, big data, and carbon emissions but also underscores the pivotal role of big data in advancing green development, offering policymakers and enterprises valuable insights and guidance to align with the Sustainable Development Goals (SDGs).

The subsequent sections of this article are structured as follows: Section “Literature review” showcases the study’s theoretical analysis and presents the research hypotheses. Section “Theoretical analysis” outlines the methods and data, encompassing model specifications, indicator measurements, and data sources. Section “Research design” elucidates the main results, encompassing baseline regression outcomes, robustness tests, and mediating regression findings. Lastly, Section “Empirical analysis” summarises the conclusions drawn from the study and offers pertinent policy recommendations.

## Literature review

Sustainable development grapples with challenges stemming from resource depletion and environmental degradation, prompting China to set forth carbon emission goals for 2030 and 2060^6^. Scholars worldwide, spanning various disciplines, have undertaken extensive research endeavours to address these pressing issues. Among these, green innovation emerges as a pivotal focus area. Intriguingly, the Green Low-Carbon Innovative Development (GLID) concept signifies a developmental paradigm that emphasises socioeconomic progress and environmental preservation. Some researchers aim to elucidate pathways toward enhanced levels of sustainable development efficiency through GLID^6^. Additionally, the integration of green development with the financial sector has garnered attention. Studies examining green finance and corporate green innovation suggest that green finance stimulates corporate green innovation by reflecting internal corporate quality and attracting external innovation resources^7^. On top of that, scholars have further categorised green innovation into green process innovation and green product innovation, positing that green process innovation positively influences green product innovation, both of which contribute to firm performance enhancement^8^. Building upon these insights, subsequent research delves into the specific diffusion pathways of these impacts, indicating that while green process innovations directly enhance economic performance, green product innovations exert an indirect influence^9^.

Contemporary societal integration across diverse fields has spurred an expanding array of studies within the realm of green initiatives. Likewise, investigations encompassing green suppliers have been integral to sustainability and green innovation performance analyses^10^. Other experts have linked stakeholder pressure, green dynamic capabilities, and green innovation to the performance of emerging small- and medium-sized enterprises (SMEs), revealing that stakeholder pressure positively influences green innovation by fostering green dynamic capabilities, ultimately enhancing firm performance^11^. Moreover, the evolution of digital technologies intertwines closely with the green domain. Studies exploring the relationship between green information and communication technology (ICT) and carbon emissions indicate that green ICT innovations can mitigate carbon emissions by enhancing energy efficiency and productivity^12^. Equally, environmental regulation serves as another pertinent perspective in the study of green innovation. Researchers have categorised green innovation into substantive and symbolic categories based on motivation, finding that environmental regulation significantly bolsters green innovation but exerts a weakened influence on substantive and symbolic green innovation^13^. However, scholars also investigate the impact of environmental regulation on urban green transformation, asserting that its effect on green technology innovation varies depending on regional economic development levels^14^.

## Theoretical analysis

### The measurement of green innovation

Several scholars offer varying definitions of green innovation, with some narrowly defining it as hardware or software innovations aimed at reducing pollution and conserving resources, while others adopt a broader perspective, encompassing any innovation that enhances energy efficiency and environmental quality^15^. A review of the literature reveals two primary approaches to measuring green innovation: one assesses green innovation performance based on inputs, desired outputs, and undesired outputs, while the other quantifies the level of green innovation using the number of green patents. The emergence of green patents as a metric stems from the urgent need to address climate change, prompting initiatives such as the World Intellectual Property Organisation’s Green List for patent classification, which scholars have increasingly utilised to gauge the extent of green innovation^16^.

### The measurement of carbon emission

Carbon emissions, as pivotal contributors to climate change, hold significant international importance, prompting various countries, including China, to devise strategies for carbon reduction, such as setting carbon neutral and carbon peaking targets. Consequently, scholars worldwide have embarked on numerous studies examining the nexus between carbon emissions and economic factors. Similarly, some scholars have investigated the influence of the digital economy on carbon emissions^3,17^, while others explore the impact of the green economy^8,9^. Divergent views exist regarding the measurement of carbon emissions, with some scholars opting for metrics like carbon intensity and per capita carbon emissions^18^, while others employ the DEA model to assess carbon emission performance^3,8,9,18,19^.

### The relationship between carbon emission and green innovation

Green innovation, operating at the technological forefront, enhances sustainability by optimising resource utilisation and mitigating environmental pollution. It also fosters enterprise-level industrial structural refinement and resource allocation optimisation, pivotal for overall operational enhancement. The impact of green innovation on carbon emissions is particularly significant, especially in pollution-intensive industries, where the adoption of low-carbon products and green technologies can curtail emissions. Similarly, green innovations in processes and equipment can bolster resource transformation efficiency, reducing production-related emissions^16^. At the managerial level, aligning with the dual carbon target, regions and enterprises have prioritised green innovation strategies, upgrading product development, production processes, and technological equipment to embrace environmentally friendly practices, thus facilitating low-carbon development^20^. Numerous scholars have explored the nexus between green innovation and urban carbon efficiency, affirming a positive correlation, with environmental regulation and industrial upgrading reinforcing this relationship^21^. Additionally, studies investigating the relationship between green technology innovation, carbon efficiency, and the transmission mechanism reveal that environmentally relevant green technology innovation enhances carbon efficiency, with economic development and urbanisation acting as mediators and financial development as a positive moderator^22^. Based on these abovementioned discussions, this study tests the following hypotheses:

#### Hypothesis 1

Green innovation attenuates regional carbon emissions.

### Green innovation, big data, and carbon emissions

Since antiquity, diverse methods have been employed to record data, permeating all aspects of life. With the advent of computers, data proliferation surged, culminating in the emergence of big data, characterised by its five defining features—value, volume, variety, veracity, and velocity^5^. These attributes are integral in the digital age, where adept handling of vast data volumes distinguishes industry frontrunners. Scholarly attention has centred on the influence of big data technology on green innovation, with a spectrum of considerations. While some researchers focus on the role of big data analysis in fostering green innovation, their concerns vary. While focusing on the role of big data analysis ability in promoting green innovation and enhancing enterprises’ competitive edge, others dissect the components of big data analysis ability—ranging from talent and business analysis to management capabilities^5^. Certain studies construct models, like the “intention-behaviour” model, grounded in organisational decision theory and planned behaviour theory, to probe the influence of big data capability on green process innovation, with green innovation intention serving as a mediating factor^4^.

Taking a contrasting viewpoint, one must inquire: “Does green innovation impact big data?” A review of existing literature reveals a consensus among scholars that big data can indeed impact green innovation, with various perspectives explored to dissect the mechanisms and pathways of this interaction. However, considering the materialist dialectic concerning the interconnectedness and mutual influence of entities, it follows that a reciprocal relationship should exist between green innovation and big data. Green innovation encompasses technological advancements facilitating pollution control, recycling, and waste minimisation, thus also fostering high-tech innovations. These green technological innovations also contribute to high-tech innovations. Notably, big data technologies can be influenced by green innovation, particularly as the world increasingly emphasises sustainable development. A prime area where green innovation exerts a notable influence is in the realm of big data technology, giving rise to environmentally favourable advancements. For instance, the adoption of cloud computing, a popular big data technology, reduces energy consumption and waste by enabling users to access data and software via the Internet instead of maintaining hardware. In like manner, innovations like the Internet of Things (IoT) hold promise in reducing energy consumption and waste, showcasing the profound impact of green innovation on big data technology.

Furthermore, big data technologies wield the potential to enhance the efficiency of innovation factor flows, bolster the application of innovative technologies within enterprises, and synergistically affect energy inputs, carbon emissions, and green emission reduction technologies, thereby fostering carbon emission reduction^20^. Big data capabilities, delineated into three components—big data collection, analysis, and insight capabilities^4^—enable companies to glean real-time market insights and integrate service capabilities, enhancing productivity while mitigating production costs and risks^23^. Some notable scholars have delved into the analytical c prowess of big data, categorising it into talent, management, and business analytics capabilities, and affirming its role in reducing industrial waste and alleviating strain on natural resources when harnessed by manufacturing firms^5^. Notably, big data serves not only as an innovation catalyst but also assumes a leadership role in green research and development (R&D) and low-carbon development^4^. Guided by this analysis, we test the following hypotheses:

#### Hypothesis 2

Big data technology acts as a mediator between green innovation and carbon intensity.

#### Hypothesis 3

Green innovation influences the trajectory of big data technology.

## Research design

### Research method

We construct a benchmark econometric model, following the approach utilised by Dong et al.^18^, to substantiate the impact of green innovation on carbon emissions. The model is formulated as follows:

$$ \ln \,Carbin_{it} = \alpha_0  {+ }\alpha_1 \ln \,Greinapp_{it} + \alpha_{2}\,controls_{it} + u_i + v_t + \varepsilon_{it} $$

where $\ln Carbin_{it}$ represents the carbon emission intensity, ${\text{l}} {\text{n}}\, Greinapp_{it}$ signifies green innovation, $controls_{it}$ denotes a series of control variables, $u_i$ denotes the individual fixed effect, $v_t$ signifies the time fixed effect, and $\varepsilon_{it}$ represents the residual term. In this model, the subscript $i$ denotes a province, and the subscript $t$ denotes time.

### Variables description

#### Explained variable

Carbon emission intensity ($\ln Carbin$): This variable delineates the relationship between the regional economic activity and carbon emissions. As per the measurement approach employed by Jing et al.^21^, carbon emission intensity is computed by dividing the carbon emissions of each province by its gross domestic product (GDP).

#### Explanatory variable

Green innovation ($\ln Greinapp$): In analysing the impact pathway of big data on the green innovation of listed companies, scholars often utilise the number of green patent applications as a metric for gauging the level of green innovation^16^. This study therefore adopts green patents as the measure for this variable, specifically the count of green inventions acquired within a given year. To handle instances where certain regions report zero green inventions, the data undergoes a logarithm transformation, with an offset of one added to all values.

#### Mediating variable

Big data ($bd$): This variable serves as a composite measure of the level of big data development within a city. Following the approach outlined in Xiao et al.^24^, four indicators—telecommunications business volume, mobile phone subscribers, Internet users, and employment share in related industries—are utilised to gauge big data development using principal component analysis (PCA).

#### Control variables

Control variables ($controls$): To bolster the precision of the regression model, a set of control variables is included. Drawing from prior research^13,14^ [27], the following control variables are incorporated: (1) Environmental regulation intensity ($eri$), derived from sulphur dioxide, wastewater, and smoke and dust emissions in the region through PCA^13,14^. (2) Industrial structure ($adsecinratio$) represented by the ratio of value added in the secondary sector to GDP^24^. (3) Urban population ($pop$), quantified by the area’s current-year resident count. (4) Natural growth rate ($natgrorate$), approximated by the region’s current-year natural birth rate. Refer to Table 1 for specific variable measurements.

**Table 1:** Variable descriptions.

Variable types	Variable	Label	Description
Explained variable	lnCarbin	Carbon emission intensity	The proportion of carbon emissions to their GDP by province
Explanatory variable	lnGreinapp	Green innovation	The logarithm of the number of green patent applications plus one
Mediating variable	bd	Big data	Four indicators measure the total number of telecommunications services, the number of mobile phone subscribers, and the number of international Internet subscribers, as well as the proportion of people employed in the relevant industry
Control variables	eri	Environmental regulation intensity	The environmental regulation intensity is calculated using sulphur dioxide emissions, wastewater emissions, and smoke and dust emissions
	adsecinratio	Industrial structure	It is the secondary industry value added as a proportion of GDP
	pop	Population	Population size of the country by province
	natgrorate	Natural growth rate	The natural growth rate of the country by province
	Province	Province	Province is a province dummy variable
	Year	Year	The year is the annual dummy variable

### Data sources and descriptions

This study utilises data spanning 30 provinces in China from 1990 to 2020. Besides, carbon emissions and GDP data are sourced from the China Stock Market & Accounting Research (CSMAR) database, while green innovation data is obtained from the China National Intellectual Property Office. The mediating variable data is extracted from the City Statistics Yearbook, and the control variables data primarily originates from the China City Statistical Yearbook. More so, the descriptive statistics of the variables are outlined in Table 2.

**Table 2:** Descriptive statistics of variables.

Variable	Obs	Mean	Std.Dev	Min	Max
lncarbin	833	− 8.078	1.021	− 12.73	− 5.509
lngreinapp	833	5.320	2.210	0	10.38
adsecinratio	833	449.7	265.5	15.83	1064
pop	833	3935	2766	171.5	11,435
natgrorate	833	61.18	53.67	− 126.8	356.9
eri	833	3.21e − 10	0.558	− 2.387	2.771
bd	833	2.54e − 10	1.018	− 1.017	16.37

## Empirical analysis

### Baseline regression results

**Figure 1:**
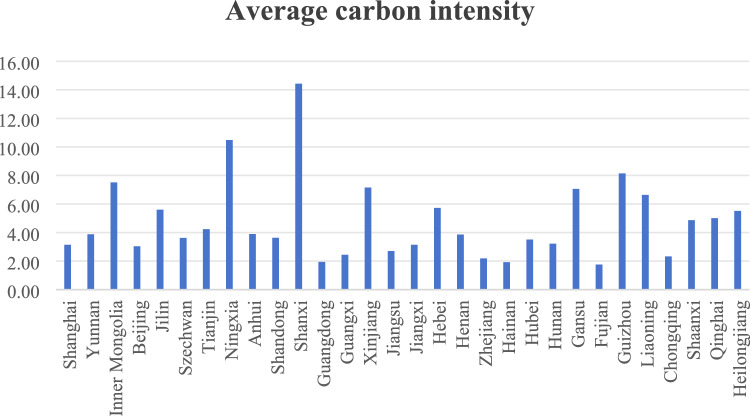
10,000 times the average of carbon emissions intensity by province.

**Figure 2:**
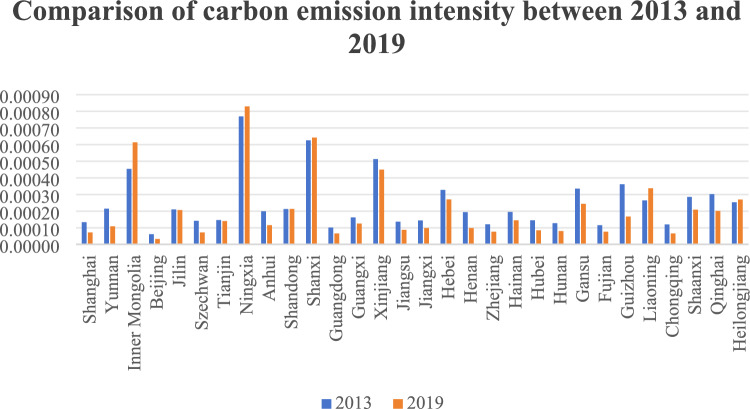
Comparison of carbon emission intensity by province in 2013 and 2019.

As an important indicator of the balanced relationship between economic development and environmental protection in a region, the value of carbon emission intensity is influenced by both carbon emissions and GDP in each province. Among the 30 provinces in Fig. 1, Shanxi, Ningxia Hui Autonomous Region and Guizhou rank among the top three in terms of carbon emission intensity, with 14.43, 10.48 and 8.14, respectively, accounting for 10 per cent, 7.4 per cent and 5.7 per cent of the total. The reason why the carbon emission intensity of these three provinces is so high is related to their industrial structure dominated by heavy industries such as coal on the one hand, and the other hand to their relatively low level of economic development and the fact that the efficiency of energy use needs to be improved. Figure 2 shows the comparison of carbon emission intensity between 2013 and 2019. The year 2013 is chosen as the starting point for the comparison because it was the year when the Chinese government organised the National Low Carbon Day for the first time, marking the beginning of China’s incorporation of the concept of low-carbon development into its national development strategy. The year 2019 was chosen because, in December of that year, the Xin Guan epidemic began to break out, after which China began to strengthen control and entered a special period. As can be seen from the comparison chart, except for Inner Mongolia, Ningxia, Shanxi, Liaoning and Heilongjiang, which are predominantly heavy industries such as coal and are sparsely populated and relatively backward in terms of economic development, the carbon emission intensity of all other provinces has shown a significant decline. This shows that, driven by the National Low Carbon Day, more and more provinces are beginning to pay attention to low-carbon development and are actively taking measures to reduce their carbon emission intensity.

**Figure 3:**
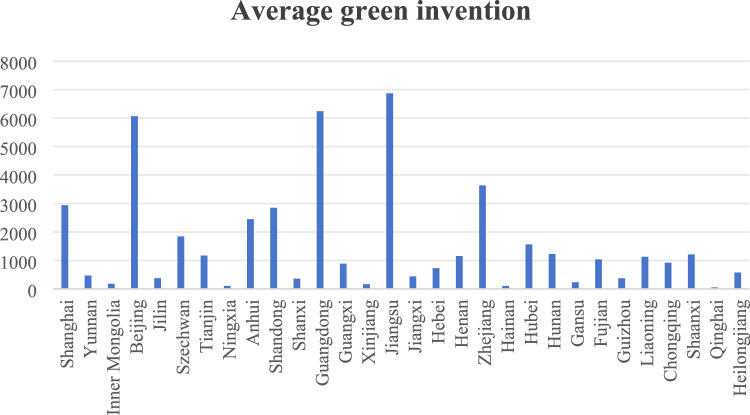
Average green invention by province.

The difference in the number of green inventions is obviously inseparable from the development of each province. In delving deeper into this phenomenon, it is not difficult to find that it is closely related to a number of factors, such as the level of economic development, the strength of scientific and technological innovation, the strength of policy support, and the awareness of environmental protection in each place. Among the 30 provinces shown in Fig. 3, Jiangsu Province, Guangdong Province and Beijing Municipality top the list of green inventions, with 6,869, 6,245 and 6,066 inventions respectively, accounting for 14%, 13% and 12% of the overall number. This data not only reflects the excellent performance of these three provinces and cities in green inventions, not only highlights their leading position in the field of green science and technology innovation, but also provides strong support for China to achieve green development and build an ecological civilisation, as well as reduces the intensity of carbon emissions and achieves the coordinated development of the economy and the environment.

After a detailed analysis of the underlying data through the core variables, a more in-depth data analysis was carried out through the constructed baseline model. The analysis commences with a variance inflation factor (VIF) test aimed at confirming the absence of multicollinearity among the variables. Table 3 presents the outcomes of the VIF test, revealing that all variables exhibit VIF values considerably below 10, indicating the absence of multicollinearity among them. Subsequently, Table 4 showcases the correlation coefficients among the observed variables. Thus, the correlation coefficient matrix underscores a significant correlation existing between the core explanatory variables and other explanatory variables, thereby bolstering the regression analysis in the empirical investigation.

**Table 3:** Variance inflation factor (VIF) test result.

Variable	VIF	1/VIF
pop	5.070	0.197
adsecinratio	4.660	0.214
eri	2.220	0.450
bd	2.070	0.484
lngreinapp	2.070	0.484
natgrorate	1.750	0.573
Mean	VIF	2.970

**Table 4:** Correlation coefficient of variables.

	lncarbin	lngreinapp	adsecinratio	pop	natgrorate	eri	bd
lncarbin	1						
lngreinapp	− 0.708***	1					
adsecinratio	− 0.0120	0.199***	1				
pop	− 0.274***	0.410***	0.840***	1			
natgrorate	0.0490	− 0.060*	0.593***	0.451***	1		
eri	0.096***	0.0460	0.672***	0.681***	0.346***	1	
bd	− 0.513***	0.566***	0.513***	0.560***	0.349***	0.299***	1

***, **, and * denote significance at the 1%, 5%, and 10% levels, respectively.

Table 5 presents the estimated results regarding the influence of green innovation on carbon emission intensity. Column (1) showcases univariate regressions incorporating province-fixed effects and year-fixed effects, while Columns (2) and (3) isolate each fixed effect separately. Likewise, Column (4) presents the regression without any fixed effects. Notably, the coefficient of green innovation consistently exhibits a significantly negative value across all variations of these fixed effects. This consistent finding underscores the resilience of green innovation in mitigating carbon emission intensity, thereby providing initial support for Hypothesis 1. Subsequently, control variables are introduced into the aforementioned regressions for comparative analysis.

**Table 5:** Univariate baseline regression results.

Variable	(1)	(2)	(3)	(4)
	lncarbin	lncarbin	lncarbin	lncarbin
lngreinapp	− 0.080*** ( − 3.031)	− 0.348*** ( − 33.190)	− 0.245*** ( − 15.597)	− 0.327*** ( − 28.888)
_cons	− 6.635*** ( − 52.703)	− 6.184*** ( − 47.797)	− 5.933*** ( − 54.490)	− 6.339*** ( − 97.184)
Year	Yes	No	Yes	No
Province	Yes	Yes	No	No
N	833	833	833	833
R^2^	0.920	0.692	0.731	0.501
Adj. R^2^	0.914	0.680	0.721	0.500

***, **, and * denote significance at the 1%, 5%, and 10% levels, respectively. The figures in () indicate the standard errors.

Table 6 illustrates the results of the baseline regression incorporating control variables. Column (1) displays regression outcomes without control variables, while Column (2) presents results with control variables. In Column (1), green innovation manifests a notable inhibitory effect on carbon emission intensity, with a direct impact coefficient of -0.080. This indicates that for every 1% increase in the green innovation indicator, carbon emission intensity diminishes by 0.08%. Despite the introduction of control variables in Column (2), the coefficient of green innovation remains negative, affirming its role in contributing to carbon reduction. This finding reaffirms Hypothesis 1, underscoring the efficacy of green innovation in dampening carbon emission intensity. Firstly, through the research, development and promotion of clean energy technologies, such as solar energy and wind energy, green innovation can effectively reduce the consumption of fossil fuels, thereby lowering the emission of greenhouse gases such as carbon dioxide. Secondly, green innovation also focuses on energy saving and emission reduction in the production process, reducing carbon emissions per unit of output by optimising production processes and improving energy efficiency. In addition, green innovation advocates green consumption and a circular economy, encouraging consumers to choose environmentally friendly products and services, promoting the recycling of resources and further reducing the intensity of carbon emissions.

**Table 6:** Baseline regression result with control variables added.

Variable	(1)	(2)
	lncarbin	lncarbin
lngreinapp	− 0.080*** ( − 3.031)	− 0.106*** ( − 3.866)
adsecinratio		0.000 (1.029)
pop		− 0.000 ( − 0.631)
natgrorate		0.001* (1.737)
eri		0.154*** (3.425)
_cons	− 6.635*** ( − 52.703)	− 6.561*** ( − 50.350)
Year	Yes	Yes
Province	Yes	Yes
N	833	833
R^2^	0.920	0.921
Adj. R^2^	0.914	0.915

### Robustness testing

#### Measurement errors

Given the potential influence of measurement errors on regression outcomes, a thorough measurement error analysis is imperative. In this study, such analysis entails substituting the indicators of both independent and dependent variables. Specifically, the indicator for the independent variable, green innovation, is altered from the number of green invention applications plus one and then logged to the number of green inventions obtained plus one and then logged. Similarly, the indicator for the dependent variable, carbon intensity, is substituted with total carbon dioxide emissions. Subsequent regression analysis utilising these modified variables to further scrutinise the impact of green innovation on carbon emissions, with the results presented in Table 7. Remarkably, the coefficient in the regression of green innovation on carbon emissions remains significantly negative, indicating the robustness of the baseline regression even after altering the variable measurement. Notably, the consistent and significant sign of the explanatory variable’s coefficient post-measurement error analysis reaffirms the argument’s robustness, validating the assertion that green innovation effectively curtails carbon emissions.

**Table 7:** Robustness test of measurement errors.

Variable	(1)
	lncarbem
lngreinob	− 0.097*** ( − 4.662)
adsecinratio	0.001*** (4.863)
pop	− 0.000 ( − 1.167)
natgrorate	0.001** (2.348)
eri	0.198*** (4.358)
_cons	9.019*** (101.188)
Year	Yes
Province	Yes
N	833
R^2^	0.918
Adj. R^2^	0.911

#### Endogenous problem

The issue of endogeneity poses a potential challenge to the study’s findings. In large sample sizes, endogeneity may lead to parameter estimations that closely approximate true parameters, thereby undermining the interpretability of results. Several factors, such as measurement error, omitted variables, and reverse causality between independent and dependent variables, can contribute to endogeneity. For instance, consider a scenario where government mandates necessitate increased green innovation in response to rising carbon emissions within a region annually. To mitigate this concern, the study employs an instrumental variables approach, utilising green innovation with a one-period lag as the instrumental variable in a two stage least square (2SLS) regression. Table 8 presents the outcomes after employing instrumental variables to address endogeneity.

**Table 8:** 2SLS result.

Variable	(1)	(2)
	lngreinapp	lncarbin
L.lngreinapp	0.716*** (0.026)	
lngreinapp		− 0.146*** (0.037)
Control variables	Yes	Yes
Year	Yes	Yes
Province	Yes	Yes
_cons	1.312*** (0.124)	− 6.493*** (0.166)
N	772	772
Anderson canon. corr. LM		392.381 [*p* = 0.0000]
Cragg–Donald Wald F statistic		735.935 [*p* = 0.0000]

Table 8 illustrates that in the first stage of the 2SLS regression, the regression coefficient of the instrumental variable on the endogenous variable is significantly positive, indicating the endogeneity of the instrumental variable. Typically, there exists no correlation between the previous period of the endogenous variable and the error term of the current period, ensuring the homogeneity of the instrumental variable. Furthermore, both under-identification and weak instrumental variable tests were conducted on the instrumental variables, yielding significant results, thereby affirming the reasonableness and validity of the instrumental variables. Subsequently, the second stage regression reveals that the coefficient of green innovation remains significantly negative, reinforcing the argument’s robustness that green innovation effectively curbs carbon emission intensity.

### Empirical testing of the mediating effect

**Figure 4:**
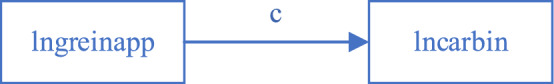
The direct impact of the mediating effect.

**Figure 5:**
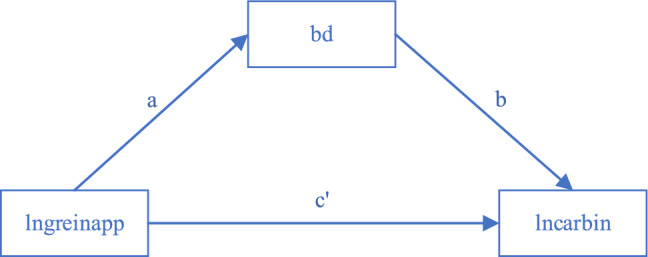
The indirect effects of the mediating effect.

This study employs a step-by-step regression to examine the significance of the coefficients on the large dataset as mediating variables sequentially. Comprising three steps in total, this testing approach elucidates the mechanism of effect, as depicted in Figs. 4 and 5 below. Initially, the regression of the independent variable on the dependent variable assesses the significance of the direct effect regression coefficient $c$. Subsequently, the regression of the independent variable on the mediating variable gauges the significance of the regression coefficient $a$. To conclude, the regression of both the independent and mediating variables on the dependent variable evaluates the significance of the regression coefficients $c^{\prime}$ and $b$.

Figure 5 illustrates the indirect effects of the mediating effect. The stepwise regression outcomes for the mediating effect are presented in Table 9, with Column (1) indicating the regression result for the direct effect of the mediating effect, Column (2) representing the regression result for the explanatory variable on the mediating variable, and Column (3) displaying the regression result for the indirect effect of this effect. The table reveals that the estimator $c$ is  − 0.106, the estimator $a$ is 0.177, the estimator $b$ is  − 0.075, and the estimator $c^{\prime}$ is  − 0.092, with all these estimators significant at the 1% confidence interval level. Their effects conform to the equation:

$$ c = c^{\prime} + a \times b $$

**Table 9:** Mediating effect results.

Variable	(1)	(2)	(3)
	lncarbin	bd	lncarbin
lngreinapp	− 0.106*** ( − 3.866)	0.177*** (3.455)	− 0.092*** ( − 3.386)
adsecinratio	0.000 (1.029)	− 0.000 ( − 0.444)	0.000 (0.976)
pop	− 0.000 ( − 0.631)	0.000*** (5.163)	0.000 (0.093)
natgrorate	0.001* (1.737)	0.000 (0.464)	0.001* (1.818)
eri	0.154*** (3.425)	− 0.087 ( − 1.033)	0.147*** (3.308)
bd			− 0.075*** ( − 3.935)
_cons	− 6.561*** ( − 50.350)	− 1.440*** ( − 5.880)	− 6.668*** ( − 50.534)
Year	Yes	Yes	Yes
Province	Yes	Yes	Yes
N	833	833	833
R^2^	0.921	0.720	0.923
Adj. R^2^	0.915	0.698	0.917

**Figure 6:**
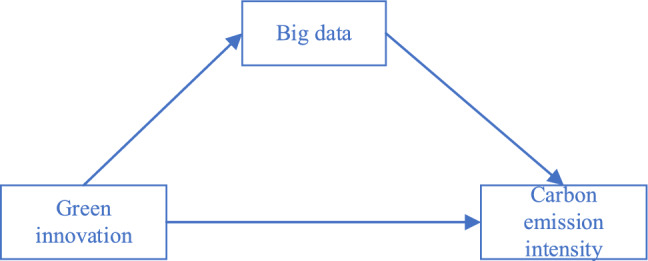
Mechanisms for mediating the effect of big data.

Furthermore, the magnitude of the mediating effect is measured by the mathematical equation: $a \times b$. This specifies the mediating mechanism of big data as depicted in Fig. 6, where green innovation directly weakens carbon intensity, while indirectly mitigates it by fostering the development of big data. This outcome effectively validates the mediating role of big data development posited in Hypothesis 2. The big data platform can facilitate the sharing and exchange of information related to green innovation and carbon emissions, promote cooperation and synergy among different fields and industries, and jointly promote green development and low-carbon transformation. Simultaneously, the supportive role of green innovation in big data development as proposed in Hypothesis 3 is also confirmed. Green innovation can promote the integration of big data with other green technologies, thereby facilitating the application of big data technologies in the environmental field.

## Conclusions and policy recommendation

### Conclusions

Green innovation emerges as a pivotal avenue for accomplishing carbon reduction objectives and advancing the realisation of national carbon peaking and carbon–neutral targets. This study delves into the intricate relationship between green innovation and carbon emissions, leveraging data from 30 provinces across China, excluding (Xizang, Hong Kong, Macao and Taiwan) and employing year and province fixed effects models to probe into the impact of green innovation on carbon emissions intensity and the mediating role of big data. Hence, the main research can be summarised as follows:

Primarily, empirical findings gleaned from extensive research endeavours furnish compelling evidence that green innovation exerts a positive influence on carbon emission reduction. These outcomes underscore the transformative potential of innovative green technologies and practices in propelling environmental sustainability endeavours. To ensure the reliability of these conclusions, encompassing measurement error examinations and endogeneity assessments, were conducted. On top of that, the successful verifications of these tests further fortified the credibility of the regression results obtained from analysing the relationship between green innovation and carbon emissions.

Secondly, through a mediation effect test, the findings underscore the pivotal role of big data as a mediator in the impact of green innovation on carbon emissions. This mediation occurs as green innovation propels the advancement of big data technologies, thereby effectively curbing carbon intensity. Analogously, the imperative for green innovation drives the evolution of environmentally sustainable trajectories for big data technologies, resulting in substantial reductions in carbon intensity.

Thirdly, the revelation that big data serves as a mediating factor in the nexus between green innovation and carbon emissions signifies the significant influence of green innovation on the development of big data. Equally, empirical evidence highlights the crucial role of green innovation in fostering the progression of big data technologies. The imperatives of green innovation, emphasising resource conservation and ecological sustainability, have catalysed the ongoing advancement of big data technologies towards resource integration and informed decision-making.

Overall, compared with previous studies on special regions such as the Yangtze River Economic Belt and those based on a spatial perspective^6,12,19,22^, this paper focuses on China and explores the role of green innovation in influencing the intensity of carbon emissions with a long time span, and also takes big data as a mediating variable to explore the mechanism of the role of big data in the process from a new perspective.

However, this study possesses certain limitations. Firstly, it does not extensively explore the role of green innovation in facilitating the development of big data. Secondly, the analysis solely focuses on the relationship between green innovation and carbon emission intensity across different provinces, leaving scope for future research to delve into potential spatial disparities in this relationship.

### Policy recommendations

To advance the attainment of carbon peaking and carbon neutrality objectives while bolstering green innovation capacity, carbon emission reduction performance, and big data development, this study proffers the following recommendations. Firstly, in terms of government policy, governments should allocate funding for the research and development of green technologies. Such initiatives can catalyze innovation and stimulate the emergence of novel environmentally friendly products and technologies. Additionally, the government can offer subsidies to companies that embrace green innovations, thereby incentivising the adoption of these pioneering technologies. Moreover, governmental intervention could be instrumental in establishing a market for green innovations through the implementation of carbon pricing mechanisms or the introduction of carbon emissions trading programs.

Secondly, from the corporate perspective, corporate managers can motivate employees to engage in green innovation research by offering incentives such as technological innovation bonuses. Furthermore, enterprises should actively integrate big data technology into their operations to effectively collect, analyze, and utilise diverse data resources for optimal resource allocation and enhanced economic efficiency. What’s more, fostering collaboration and knowledge-sharing through public service platforms can encourage innovation and facilitate the timely identification of challenges and opportunities for improvement. Congruently, in the realm of education, schools can actively participate in collaborative innovation initiatives with research institutions or private enterprises, thereby fostering industry-academia-research partnerships and ensuring the accessibility and practicality of green innovation technologies. Moreover, educational institutions can expand their array of general education programs to disseminate knowledge about green innovation among students, heighten their awareness of green innovation, and nurture a larger pool of talent in this domain. Above and beyond, the government should strive to cultivate a green and digitally-driven environment, enhance green innovation performance, promote carbon reduction initiatives, and work towards achieving the objectives of carbon peaking and carbon neutrality.

In conclusion, this study underscores the critical role of green innovation and big data technologies in curbing carbon emissions and advancing towards carbon peaking and neutrality goals. Through empirical analysis and mediation testing, it has been established that green innovation not only directly contributes to carbon emission reduction but also exerts an indirect influence through its facilitation of big data development. Governmental policies supporting research funding and incentivising green innovation adoption, alongside corporate integration of big data technologies, are recommended pathways towards achieving sustainable environmental outcomes. Education therefore plays a pivotal role in nurturing talent and fostering collaboration in green innovation initiatives. Ultimately, concerted efforts at the governmental, corporate, and educational levels are imperative to propel society towards a greener and digitally-driven future, aligned with carbon reduction objectives.

A recommendation stemming from this study suggests that policymakers should prioritise allocating resources for green innovation research and incentivising its adoption while encouraging the integration of big data technologies across industries to enhance resource efficiency and carbon reduction efforts. However, it’s crucial to acknowledge limitations, such as the focus on provincial-level data in China, which may not fully capture regional nuances. Future studies could expand this analysis by incorporating data from other regions and exploring the longitudinal effects of green innovation and big data technologies on carbon emissions. Additionally, investigating the role of other factors, such as governmental regulations and market dynamics, could provide a more comprehensive understanding of the mechanisms driving carbon reduction initiatives.

## Data Availability

The datasets used and/or analysed during the current study are available from the corresponding author upon reasonable request.
